# Digital phenotyping by consumer wearables identifies sleep-associated markers of cardiovascular disease risk and biological aging

**DOI:** 10.1038/s42003-019-0605-1

**Published:** 2019-10-04

**Authors:** Jing Xian Teo, Sonia Davila, Chengxi Yang, An An Hii, Chee Jian Pua, Jonathan Yap, Swee Yaw Tan, Anders Sahlén, Calvin Woon-Loong Chin, Bin Tean Teh, Steven G. Rozen, Stuart Alexander Cook, Khung Keong Yeo, Patrick Tan, Weng Khong Lim

**Affiliations:** 1 SingHealth Duke-NUS Institute of Precision Medicine, National Heart Centre Singapore, Singapore, Singapore; 2 Cardiovascular and Metabolic Disorders Program, Duke-NUS Medical School, Singapore, Singapore; 3 National Heart Research Institute Singapore, National Heart Centre, Singapore, Singapore; 4 Department of Cardiology, National Heart Centre, Singapore, Singapore; 5 Department of Medicine, Karolinska Institutet, Karolinska, Sweden; 6 Cancer and Stem Biology Program, Duke-NUS Medical School, Singapore, Singapore; 7 Laboratory of Cancer Epigenome, Division of Medical Sciences, National Cancer Centre, Singapore, Singapore; 8 Institute of Molecular and Cell Biology, Agency for Science, Technology and Research, Singapore, Singapore; 9 Cancer Science Institute of Singapore, National University of Singapore, Singapore, Singapore; 10 Centre for Computational Biology, Duke-NUS Medical School, Singapore, Singapore; 11 National Heart and Lung Institute, Imperial College London, London, UK; 12 MRC Clinical Sciences Centre, Imperial College London, London, UK; 13 Biomedical Research Council, Agency for Science, Technology and Research, Singapore, Singapore

**Keywords:** Predictive markers, Risk factors, Data integration, Senescence

## Abstract

Sleep is associated with various health outcomes. Despite their growing adoption, the potential for consumer wearables to contribute sleep metrics to sleep-related biomedical research remains largely uncharacterized. Here we analyzed sleep tracking data, along with questionnaire responses and multi-modal phenotypic data generated from 482 normal volunteers. First, we compared wearable-derived and self-reported sleep metrics, particularly total sleep time (TST) and sleep efficiency (SE). We then identified demographic, socioeconomic and lifestyle factors associated with wearable-derived TST; they included age, gender, occupation and alcohol consumption. Multi-modal phenotypic data analysis showed that wearable-derived TST and SE were associated with cardiovascular disease risk markers such as body mass index and waist circumference, whereas self-reported measures were not. Using wearable-derived TST, we showed that insufficient sleep was associated with premature telomere attrition. Our study highlights the potential for sleep metrics from consumer wearables to provide novel insights into data generated from population cohort studies.

## Abstract (web summary)

Jing Xian Teo et al. used sleep tracking data from consumer wearables, together with phenotypic data from 482 volunteers to identify factors associated with habitual sleep duration and sleep efficiency. They identified sleep metrics associated with cardiovascular disease risk markers, such as body mass index, and linked insufficient sleep to premature telomere shortening, a biomarker of aging.

## Introduction

The relationship between sleep and various health outcomes has been extensively studied. Among others, insufficient sleep has been linked to obesity^1,2^, hypertension^3–6^, cardiovascular disease (CVD)^7–10^, insulin resistance^11–14^, and even premature death^15,16^. Previous studies on sleep-health interactions have relied on three methods to quantify sleep; sleep questionnaires/diaries, actigraphy, and polysomnography (PSG). There are drawbacks associated with each approach. First, sleep questionnaires/diaries lack precision and rely on subjective recall^17^. Second, actigraphy involves specialized devices and are only suitable for relatively short studies. Finally, PSG studies, while being the gold-standard in accuracy, are very resource-intensive to conduct^18^.

The digital revolution has resulted in the proliferation of consumer wearables with activity tracking functionality. These devices range from relatively simple and low-cost fitness trackers to more sophisticated and multifunctional smartwatches. Beyond physical activity, such devices also track sleep duration and sleep stages, the latter using integrated heart rate (HR) sensors. Although marketed as tools to promote healthy sleep habits, the rapidly growing adoption of consumer wearables suggest their potential as sources of quantitative sleep data for sleep-related biomedical research.

Recent studies have begun exploring the potential of sleep data derived from consumer wearables. First, researchers have compared the accuracy of sleep as measured by consumer wearables from several manufacturers (e.g., Fitbit and Jawbone) with gold standard PSG measurements^19–22^. Consumer wearables were found to perform similarly to actigraphs in that they were accurate in detecting sleep but did less well in detecting wake^21,23^. Some cohort studies have begun using consumer wearables. For example, we recently used Fitbit-derived sleep tracking data to show differences in sleep patterns among volunteers stratified into various activity pattern clusters^24^. Xu et al. used Fitbit Charge HR sleep data from 748 individuals to demonstrate independent associations between both sleep duration and sleep duration variation with body mass index (BMI)^25^. Additionally, Turel et al. used Fitbit devices to show a negative association between sleep duration and abdominal obesity^26^.

Despite these advances, the potential role of sleep metrics from consumer wearables in population health studies remains largely unexplored. First, there has been limited comparison between sleep metrics from consumer wearables and self-reported sleep quality from questionnaires such as the Pittsburgh Sleep Quality Index (PSQI), which is typically used in large cohort studies where it is it impractical and costly to use actigraphy or PSG^25,27^. This is important if consumer wearables are to replace or augment sleep questionnaires in future cohort studies. Second, the utility of consumer wearables in identifying associations between sleep and health markers is relatively unknown, especially in Asians; a population with considerably different sleep behavior compared to Western cohorts^25,28^. Health markers of typical interest in population health studies include CVD risk markers such as anthropometrics, blood pressure, lipid profile, and fasting blood glucose (FBG). Telomeres are hexameric repeats that cap chromosome ends and are progressively shortened with successive cell divisions^29^. Leukocyte telomere length (LTL) is thus usually included in cohort studies as a biomarker of aging^30^. Finally, there has yet to be an exploration of how wearable sleep data correlates with demographic, socioeconomic, and lifestyle factors.

Using an expanded cohort and dataset compared to our initial study^24^, we sought to address these gaps through a comprehensive analysis of sleep data obtained from Fitbit Charge HR activity trackers worn by 482 Singaporean volunteers. Apart from the wearable tracking, these volunteers were comprehensively profiled for CVD risk markers and LTL. We found that sleep metrics from consumer wearables could be used to identify not just sleep-related demographic, socioeconomic, and lifestyle factors in health cohorts, but also CVD risk markers affected by sleep duration and quality. Furthermore, we used wearable-derived sleep duration to show that volunteers with insufficient sleep experienced premature telomere shortening. Our results highlight the potential for consumer-grade wearables as sources of quantitative sleep metrics in population health studies, thus increasing power to detect sleep-associated factors.

## Results

### Comparison between wearable-derived and subjective sleep metrics

**Fig. 1:**
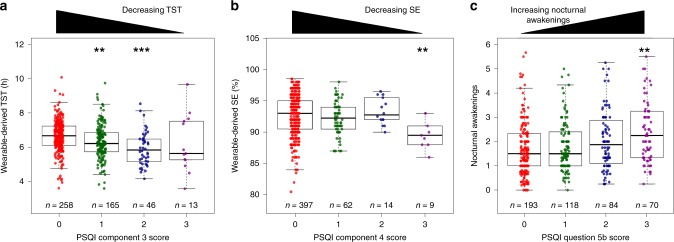
Comparison between wearable-derived and self-reported sleep metrics. **a** Wearable-derived TST and PSQI Component 3 score (sleep duration). **b** Wearable-derived SE and PSQI Component 4 score (sleep efficiency). **c** Wearable-derived nocturnal awakenings and PSQI Component 5b score (nocturnal awakenings). Asterisks denote significance of component score in linear model compared to reference score of 0. *p < 0.01, ***p < 0.001. TST total sleep time, SE sleep efficiency, PSQI Pittsburgh Sleep Quality Index

The cohort of 482 volunteers was tracked using Fitbit Charge HR wearables that measured physical activity, HR, and sleep. Summary statistics for the cohort are shown in Table 1. The volunteers were on average 46 years of age (range 21 to 69 years) at enrollment. On average, they had 4 nights of tracked sleep (range 3 to 11 nights), with a mean total sleep time (TST) of 6 h and 28 min. We first compared objective sleep measures from consumer wearables to subjective PSQI responses. The PSQI sleep questionnaire comprises several components, each encompassing a different aspect of sleep quality. It then summarizes individual component scores into a global PSQI score. We compared wearable-derived TST and SE with global PSQI scores and found correlations in neither (*r*_s_ = −0.089, *p* = 0.091 and *r*_s_ = −0.080, *p* = 0.129 respectively). Wearable-derived TST, however, showed a significant, albeit weak correlation with self-reported TST (*r*_s_ = 0.283, *p* = 2.394E-10). We asked if this weak correlation could be due in part to the relatively short study duration, and therefore modified the inclusion criteria from at least three nights of tracked sleep to four and five nights. Indeed, when the thresholds increased, correlation with self-reported TST rose to 0.322 (*p* = 6.218E-09, *n* = 310) and 0.397 (*p* = 1.425E-06, *n* = 138) respectively. We then categorized self-reported TST by levels specified in component 3 of the PSQI, which profiles habitual sleep duration. Compared to those with the lowest score of 0 (>7 h of sleep), those with scores of 1 (6 to7 hours) and 2 (5 to 6 h) had lower wearable-derived TST (*β* = −0.321, CI = −0.512 to −0.131, *p* = 0.001 and *β* = −0.721, CI = −1.027 to −0.415, *p* = 4.94E-06 respectively, Fig. 1a). However, those with a score of 3 (<5 h) exhibited no significant difference in TST compared to those with a score of 0 (*β* = −0.428, CI = −0.972 to 0.115, *p* = 0.123), indicating lower concordance with wearable-derived TST among those with self-perceived chronic sleep deprivation. This may be due to the limited number of volunteers in that category (*n* = 13) and correspondingly higher variability in wearable-derived TST. Overall, volunteers on average over-estimated habitual sleep duration by 6 min compared to objective wearable-derived measurements (*p* = 0.067, paired Student’s *t*-test).

**Table 1:** Summary statistics of study volunteers

Characteristic	Female (*n* = 262; 54.36%)	Male (*n* = 220; 45.64%)	Test
Age, years	46.21 (11.35)	45.80 (12.70)	0.703
Ethnicity			0.001
Chinese	247 (94.3)	192 (87.3)	
Indian	6 (2.3)	11 (5.0)	
Malay	2 (0.8)	14 (6.4)	
Others	7 (2.7)	3 (1.4)	
BMI, kg/m^2^	22.83 (3.80)	24.27 (3.06)	<0.001
WC, cm	79.01 (11.08)	86.82 (9.36)	<0.001
WHtR	0.50 (0.07)	0.51 (0.05)	0.073
BFP, %	33.16 (7.65)	23.64 (6.48)	<0.001
SMP, %	35.49 (4.40)	42.57 (4.25)	<0.001
SBP, mmHg	122.54 (17.53)	132.06 (15.04)	<0.001
DBP, mmHg	73.82 (12.85)	82.40 (10.88)	<0.001
Total Cholesterol, mmol/l	5.38 (0.93)	5.39 (0.97)	0.922
LDL, mmol/l	3.34 (0.81)	3.45 (0.91)	0.183
HDL, mmol/l	1.58 (0.32)	1.36 (0.32)	<0.001
TGs, mmol/l	1.02 (0.54)	1.33 (0.80)	<0.001
Glucose,mmol/l	5.18 (0.50)	5.39 (0.73)	<0.001
RestingHR, (Fitbit, bpm)	69.79 (6.37)	68.23 (6.48)	0.008
DailySteps, (Fitbit, ×1000)	10349.56 (3466.18)	11061.09 (3818.57)	0.033
LTL, bp	−47.72 (443.41)	−57.12 (536.72)	0.899
GPPAQ Score	1.31 (1.12)	1.95 (1.11)	<0.001
Wearable-derived TST, hr	6.60 (1.00)	6.32 (0.98)	0.002
Self-reported TST (PSQI Sleep Hour), hr	6.59 (1.04)	6.56 (1.00)	0.796
Wearable-derived SE, %	93.08 (2.84)	92.00 (3.22)	<0.001
Self-reported SE (PSQI Component 4 Score), %	0.26 (0.63)	0.22 (0.56)	0.407
Wearable-derived nocturnal awakenings	2.00 (1.39)	1.96 (1.58)	0.778
Self-reported nocturnal awakenings (PSQI Question 5b)	1.02 (1.06)	1.13 (1.13)	0.263
Global PSQI Score	3.73 (2.36)	3.78 (2.14)	0.854

Test *p*-values for between gender comparisons are shown: For continuous variables, two-sided Student’s *t*-test was used, whereas categorical values were evaluated using the chi-squared test

*BMI* body mass index, *WC* waist circumference, *WHtR* waist-to-height ration, *BFP* body fat percentage, *SMP* skeletal muscle percentage, *SBP* systolic blood pressure, *DBP* diastolic blood pressure, *LDL* low-density lipoprotein, *HDL* high-density lipoprotein, *TG*  triglycerides, *LTL* leucocyte telomere length, *TST* total sleep time, *SE* sleep efficiency, *GPPAQ* General Practice Physical Activity Questionnaire, *PSQI* Pittsburgh Sleep Quality Index

Apart from TST, the Fitbit wearables also measure sleep efficiency (SE) as the fraction of TST over total time in bed. Similarly, the PSQI estimates SE using self-reported habitual sleep duration, sleep times and wake times. An overall comparison between wearable-derived and self-reported SE revealed no correlation (*r*_s_ = −0.080, *p* = 0.081). However, when self-reported SE was grouped by PSQI component 4 scoring thresholds (>85%, 75–84%, 65–75%, <65%), volunteers in the <65% group had significantly lower wearable-derived SE compared others (mean SE = 89.722% vs 92.638%, two-sided Student’s *t*-test *p*-value = 0.005, Fig. 1b). Thus, only volunteers with the poorest self-perceived SE have concordantly lower wearable-derived SE. We note that wearable-derived SE is almost uniformly high (mean SE = 92.584%), with little variation (SE standard deviation = 3.060%); possibly due to the lower sensitivity of Fitbit wearables in detecting wake states as opposed to sleep states^19^.

Another wearable-derived sleep metric available was the number of awakenings per sleep session. For each volunteer, we obtained the average daily number of nocturnal awakenings. We then compared this number against responses to question 5b of the PSQI, which asks volunteers how frequently they had trouble sleeping due to waking up in middle of the night or early morning. We observed a weak correlation (*r*_s_ = 0.189, *p* = 4.114E-05), with volunteers reporting the highest (≥3 times/week) number of nocturnal awakenings having significantly higher daily wearable-detected nocturnal awakenings compared to those reporting no trouble sleeping in the past month (*β* = 0.586, CI = 0.185 to 0.987, *p* = 0.004, Fig. 1c). Collectively, our findings show that consumer wearables provide objective sleep metrics that, although associated with to a certain extent, are orthogonal to subjective measures of sleep quality.

### Relationship between wearable sleep metrics and cohort demographics

We next determined if wearable-derived sleep metrics can identify sleep-associated demographic, socioeconomic and lifestyle factors in our cohort. These factors were obtained from responses to detailed demographic and socioeconomic questionnaires administered during volunteer recruitment.

**Fig. 2:**
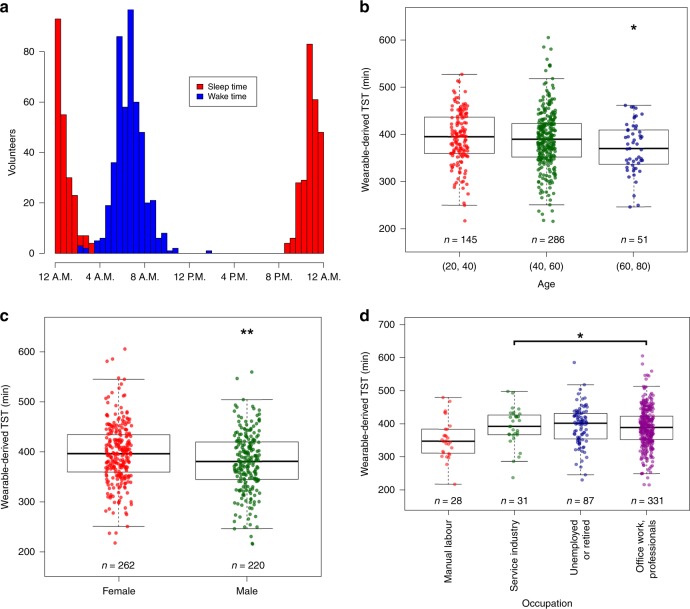
Wearable sleep duration and demographic factors. **a** Distribution of volunteer sleep and wake times. **b**–**d** Wearable-derived TST by **b** age-group, **c** gender and **d** occupation type. Asterisks denote significance of factor in linear model compared to reference level (leftmost factor). *p < 0.05, **p < 0.01, ***p < 0.001. TST total sleep time

Although the volunteers were predominantly of Chinese ethnicity (*n* = 439, 91.1%), some were of Malay (*n* = 16, 3.3%), Indian (*n* = 17, 3.5%) and other (*n* = 10, 2.1%) ethnicities. Adjusting for age and gender, we found that Malay volunteers on average slept 41 min less than Chinese volunteers (CI = −71 to −10 min, *p* = 0.009) whereas Indian volunteers slept for an additional 32 min compared to their Chinese counterparts (CI = 3 to 60 min, *p* = 0.029). Similarly, after adjusting for gender and ethnicity, we found that TST decreased with age (*β* = −0.493, CI = −0.941 to −0.044, *p* = 0.032, Fig. 2b). For gender, we found that after adjusting for age and ethnicity, female volunteers on average slept 16 min longer than their male counterparts (CI = −26 to −5 min, *p* = 0.005, Fig. 2c).

We next examined relationships between wearable-derived TST and socioeconomic factors. Among others, we considered income levels, residence type, education level and occupation type (Supplementary Table 1). Of these factors, occupation type and residence type were associated with TST. Volunteers engaged in manual work slept 27 min less then volunteers engaged in other occupation types (i.e., service industry, office work and unemployed/retired, CI = −50 to −4 min, *p* = 0.022, Fig. 2d). Furthermore, volunteers living in private residences slept 15 min longer than those living in public housing (CI = 2–27 min, *p* = 0.019).

Several self-reported lifestyle factors were also analyzed for association with wearable-derived TST. These included exercise, smoking status, alcohol consumption and caffeine consumption (Supplementary Table 2). Apart from alcohol consumption, no other significant associations were found. Volunteers who self-reported alcohol consumption within the past three months slept 19 min longer than those who did not, adjusting for age, gender and ethnicity (CI = 8–30 min, *p* = 8.54E-04). When alcohol consumption was broken down by type of alcohol, volunteers reporting consumption of hard liquor had the largest difference in TST compared to those that did not (28 min longer, CI = 10–46 min, *p* = 0.002), followed by red wine (19 min longer, CI = 5– 33 min, *p* = 0.008) and beer (18 min longer, CI = 4–32 min, *p* = 0.014). We did not identify any significant associations when the analyses in this section were repeated using self-reported TST instead of wearable-derived TST (Supplementary Tables 1 and 3).

### Wearable-derived sleep metric associations with CVD markers

A key aim of the study cohort was to study CVD risk in normal individuals. To that end, various baseline markers of cardiovascular health were collected, including anthropometric measurements (BMI, waist circumference [WC], waist-to-height ratio [WHtR], body fat percentage [BFP], skeletal muscle percentage [SMP]), resting HR, blood pressure (systolic blood pressure [SBP], diastolic blood pressure [DBP]), lipid panel results (total cholesterol [TotalChol], low-density lipoprotein [LDL], high-density lipoprotein [HDL], triglycerides [TG] and FBG). To determine if wearable-derived sleep metrics were associated with any of these CVD risk markers, we performed multiple linear regression using three different models. Models 1 and 2 consider TST and SE individually, whereas Model 3 includes both TST and SE. All three models included age, gender and daily step counts as covariates. Wearable-derived TST was associated with BMI, TotalChol and resting HR, whereas wearable-derived SE was associated with BMI, WC, WHtR and HDL levels (Table 2, results for Model 3 are in Supplementary Table 3). Neither wearable-derived TST nor SE were significantly associated with blood pressure or FBG levels in this study. As with cohort demographics, we tested self-reported sleep metrics for TST and SE using the same models and found no significant associations (Supplementary Table 4). We also considered models with interactions between wearable-derived sleep metrics and two factors; age and gender. Apart from an interaction between wearable-derived TST and age for SMP, we did not identify any other significant interactions (Supplementary Table 5).

**Table 2:** Association between wearable-derived sleep metrics and CVD risk markers

	Wearable-derived TST and SE			
Marker	Model 1^a^		Model 2^b^	
	Wearable-derived TST		Wearable-derived SE	
	β (95% CI)	*p*	β (95% CI)	*p*
BMI	−5.683E-03 (−1.111E-02 to −2.735E-04)	**0.040**	−1.089E-01 (−2.127E-01 to −5.105E-03)	**0.040**
WC	1.100E-03 (−1.499E-02 to 1.720E-02)	0.893	−4.103E-01 (−7.169E-01 to −1.036E-01)	**0.009**
WHtR	−3.750E-05 (−1.345E-04 to 5.952E-05)	0.449	−2.515E-03 (−4.364E-03 to −6.665E-04)	**0.008**
RestingHR	−1.447E-02 (−2.423E-02 to −4.721E-03)	**0.004**	−2.448E-02 (−2.132E-01 to 1.643E-01)	0.800
SBP	−8.718E-03 (−3.360E-02 to 1.616E-02)	0.493	−1.795E-01 (−6.569E-01 to 2.978E-01)	0.461
DBP	−8.249E-03 (−2.676E-02 to 1.026E-02)	0.383	−1.100E-02 (−3.665E-01 to 3.445E-01)	0.952
TotalChol	−1.493E-03 (−2.935E-03 to −5.190E-05)	**0.043**	4.961E-03 (−2.282E-02 to 3.274E-02)	0.726
LDL	−1.304E-03 (−2.624E-03 to 1.548E-05)	0.053	2.976E-03 (−2.250E-02 to 2.845E-02)	0.819
HDL	−9.582E-05 (−5.793E-04 to 3.877E-04)	0.670	9.446E-03 (2.062E-04 to 1.869E-02)	**0.046**
TG	−2.046E-04 (−1.231E-03 to 8.221E-04)	0.670	−8.832E-03 (−2.852E-02 to 1.086E-02)	0.380
FBG	−2.354E-04 (−1.165E-03 to 6.937E-04)	0.620	−2.399E-03 (−2.018E-02 to 1.538E-02)	0.792
BFP	−1.015E-02 (−2.116E-02 to 8.620E-04)	0.071	−1.729E-01 (−3.836E-01 to 3.777E-02)	0.108
SMP	6.464E-03 (-1.530E-04 to 1.308E-02)	0.056	1.030E-01 (−2.366E-02 to 2.297E-01)	0.112

Model 1 = TST only, Model 2 = SE only, Model 3 = TST + SE. All models include age and gender as covariates. *p*-values in bold are statistically significant (*p* < 0.05)

*BMI* body mass index, *WC* waist circumference, *WHtR* waist-to-height ration, *BFP* body fat percentage, *SMP* skeletal muscle percentage, *SBP* systolic blood pressure, *DBP* diastolic blood pressure, *TotalChol*  total cholesterol, *LDL* low-density lipoprotein, *HDL* high-density lipoprotein, *TG* triglycerides, *TST* total sleep time, *SE* sleep efficiency and *FBG* fasting blood glucose.

^a^Marker~Age + Gender + Ethnicity + AverageDailyTotalSteps + Wearable-derived TST

^b^Marker~Age + Gender + Ethnicity + AverageDailyTotalSteps + Wearable-derived SE

### Wearable-inferred sleep insufficiency is associated with premature telomere attrition

**Fig. 3:**
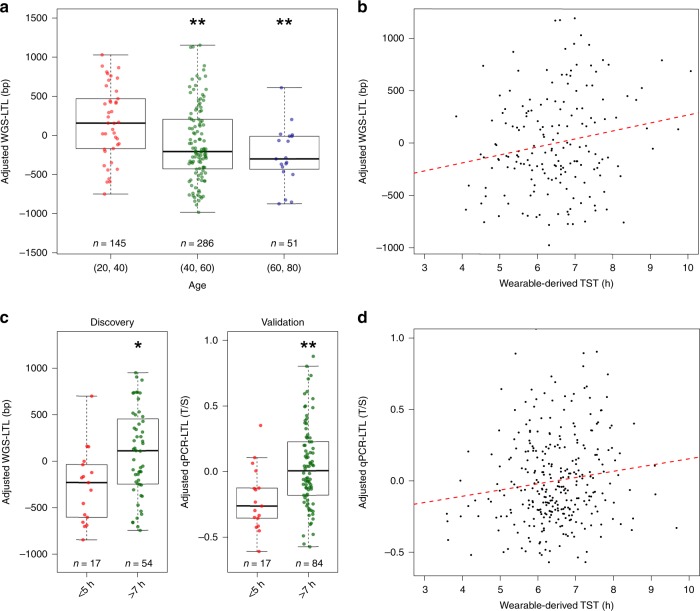
Wearable-derived TST predicts leukocyte telomere length. **a** Adjusted WGS-LTL by age-group. **b** Adjusted wearable-derived TST and adjusted WGS-LTL. **c** Adjusted WGS-LTL and adjusted qPCR-LTL of volunteers with insufficient (<5 h) and adequate (>7 h) of TST. **d** Adjusted wearable-derived TST and adjusted qPCR-LTL. Asterisks denote significance of component score in linear model compared to reference score of 0. **p < 0.01, ***p < 0.001. LTL leukocyte telomere length, WGS-LTL LTL estimated using whole-genome sequencing, qPCR-LTL LTL estimated using quantitative PCR, TST total sleep time, bp base pairs, T/S T/S ratio. All LTL values are adjusted for age, gender, ethnicity, and BMI

A subset of the cohort (*n* = 175) underwent whole-genome sequencing (WGS) for prospective genetic studies. Studies have shown that LTL can be estimated from WGS data by analyzing reads containing the telomeric repeat motif (TTAGGG). We used a tool called Telomerecat that estimates LTL by calculating the ratio between read-pairs completely mapping to the telomere and those that span the telomere boundary^31^. WGS-inferred LTL (“WGS-LTL”) was computed for the 175 volunteers with WGS data, and the estimated values corrected to account for different sequencing runs. We first selected 20 volunteers of varying WGS-LTL and experimentally measured LTL using quantitative polymerase chain reaction (qPCR, “qPCR-LTL”). LTL measurements from the two methods were significantly correlated (*r*_p_ = 0.642, *p* = 0.002). WGS-LTL also correlated with volunteer age (*r*_p_ = −0.251, *p* = 8.184E-04). When volunteers were split into age-groups of 20–40, 40–60 and 60–80 years, volunteers in the 40–60 and 60–80 years age-groups had WGS-LTL that were on average 259 base pairs (bp) and 432 bp shorter than the reference group of 20–40 years (CI = −440.101 to −78.434, *p* = 0.006 and CI = −698.315 to −166.112, *p* = 0.002 respectively, adjusted for gender, ethnicity and BMI, Fig. 3a). We then asked if wearable-derived sleep metrics were associated with telomere length and found a positive association between wearable-derived TST and WGS-LTL (*β* = 1.275, CI = 0.187–2.363, *p* = 0.023, adjusted for age, gender, ethnicity and BMI, Fig. 3b). We further examined this association by considering two groups of volunteers; those with wearable-derived TST < 5 h and those with wearable-derived TST > 7 h. Volunteers with adequate sleep (>7 h) had WGS-LTL that was on average 356 bp longer than those with insufficient sleep (<5 h) (CI = 74.573–636.538, *p* = 0.016, adjusted for age, gender, ethnicity, and BMI, Fig. 3c).

To validate this finding, we performed qPCR-based telomere length estimation on 305 volunteers from the cohort without WGS data. We were able to replicate the association between wearable-derived TST and qPCR-LTL (*β* = 7.288E-04, CI = 8.318E-05 to 0.001, *p* = 0.028, adjusted for age, gender, ethnicity, and BMI, Fig. 3d), as well as the observation that volunteers with adequate sleep had longer telomeres than those with insufficient sleep (*β* = 0.253, CI = 0.079 –0.427, *p* = 0.005, adjusted for age, gender, ethnicity and BMI). However, when self-reported TST was used instead of wearable-derived TST, a significant association with LTL was found in the WGS-based discovery cohort (β = 93.835, CI = 25.645–162.026, *p* = 0.008) but not in the qPCR-based validation cohort (*β* = 0.020, CI = −0.015 to 0.055, p = 0.258). We also did not identify any significant interactions between wearable-derived TST and two factors; age (WGS-LTL: *β* = 0.044, CI = −0.061 to 0.150, *p* = 0.413, qPCR-LTL: *β* = −4.004E-05, CI = −9.432E-05 to 1.423E-05, *p* = 0.149) and gender (WGS-LTL: *β* = −0.121, CI = −2.292 to 2.049, *p* = 0.913, qPCR-LTL: *β* = 0.001, CI = 4.947E-04 to 0.002, *p* = 0.224) in influencing LTL.

There is evidence that excessive sleep may be associated with not only increased morbidity and mortality^32^, but also shorter telomeres^33,34^. This could result in increased LTL heterogeneity in the adequate sleep group (>7 h). We thus repeated the analysis with the adequate sleep group further stratified into two groups; adequate sleep (>7 h but < = 9 h) and long sleep (>9 h), with the adequate sleep group set as reference (Supplementary Fig. 1). For both the WGS-based (*β* = −333.372, CI = −604.138 to −62.606, *p* = 0.019) and qPCR-based cohorts (*β* = −0.262, CI = −0.436 to −0.087, *p* = 0.004), the difference in LTL between the insufficient sleep and adequate sleep groups remained significant. Likewise, there was no significant difference in LTL between the adequate sleep and long sleep groups in both cohorts (WGS-based: *β* = 350.281, CI = −149.690 to 850.251, *p* = 0.175, qPCR-based: *β* = −0.201, CI = −0.571 to 0.168, *p* = 0.289), although there was a non-significant reduction in LTL in the long sleep group of the qPCR-based cohort.

## Discussion

We have shown in a sizeable cohort of 482 individuals how sleep metrics from consumer wearables can be used in biomedical research, particularly in the context of population health studies. This multi-modal cohort is one of the largest to-date with consumer wearable sleep metrics. Our comparison of objective wearable-derived sleep metrics against subjective measures obtained through the PSQI provides insights into the characteristics of these two modalities. The weak correlation between wearable-derived and self-reported TST is consistent with previous studies comparing objectively measured TST (PSG and actigraphy) with self-reported TST^35–37^. For example, Landry et al. reported a correlation of 0.29 between actigraph-derived and self-reported TST, despite a longer minimum tracking duration than ours (14 nights vs 3 nights). Concordance was poorer when we compared wearable-derived SE and number of nocturnal awakenings with their self-reported counterparts. This is again consistent with previous reports of no correlation between objectively-measured (PSG and actigraphy) and PSQI-derived SE^37,38^. Our comparison of wearable-derived sleep metrics against volunteer-provided responses to the PSQI—an instrument frequently used in population studies, will inform investigators considering using wearables in future studies. Furthermore, the limitations highlighted present opportunities for researchers to develop algorithms that can more accurately detect wake states. Indeed, newer generations of Fitbit wearables (e.g., Alta HR) combine accelerometer and HR variability data to accurately stage sleep, increasing wake detection specificity to over 88%^20^.

The questionnaire responses from volunteers allowed us to study how wearable-derived sleep metrics are influenced by demographic, socioeconomic and lifestyle factors. Among others, we showed that wearable-derived TST was associated with age, gender, ethnicity, occupation type, and even habitual alcohol consumption. With many countries realizing the importance of population health studies, the use of consumer wearables to identify demographic, socioeconomic and lifestyle factors associated with sleep duration could provide vital insights into population sub-groups at risk for poorer health outcomes due to insufficient sleep.

Our analysis on how wearable-derived TST and SE relate to various CVD risk markers demonstrate the utility of wearable-derived sleep metrics in biomedical research. Xu et al. previously described a link between habitual sleep duration estimated using Fitbit Charge HR devices and BMI in a predominantly European-American cohort of 471 individuals^25^. We identified in an Asian cohort this relationship despite a considerably shorter average tracking duration (4 nights vs 78 nights), suggesting that sleep metrics from even short studies can be useful. One novel aspect of this study was our analysis of wearable-derived SE against CVD risk markers. Our findings of links between SE and three obesity markers; BMI, WC and WHtR, is supported by previous studies performed using orthogonal approaches such as PSG and actigraphy^39,40^. This indicates that wearables can contribute beyond TST to health cohort studies, notwithstanding current limitations to the accuracy of wearable-derived SE. The paucity of associations between wearable-derived sleep metrics and clinical parameters such as FBG (a marker of insulin sensitivity), and blood pressure could be due in part to the cohort size, and highlights the need for wearables to be included in larger population-scale cohort studies in order to thoroughly assess their utility.

In both analyses of cohort demographics and CVD risk markers, no significant associations were identified when self-reported TST was used. This stood in contrast to wearable-derived TST, which showed multiple significant associations. Several of these associations have been previously described; for example, shorter TST has been linked to older age^41^, male gender^25,41^, manual labor^42^, and increased BMI^1,25^. Several factors may explain the poor performance of self-reported TST. First, wearable-derived TST is more precise than self-reported TST (minute-level vs hour-level resolution), resulting in higher sensitivity to small differences in TST. Second, self-reported TST is only moderately correlated with TST objectively measured using PSG^35,36^ or actigraphy^37^. In contrast, TST from consumer wearables (e.g., Fitbit) are highly correlated with TST measured using PSG^20,43,44^ and actigraphy^23^. Third, the subjectivity of self-reported TST exposes it to biases; one study found that while men self-reported longer TST than women, actigraphy data indicated the opposite^45^. Questionnaires and PSG are on opposite ends of the sleep detection accuracy spectrum^17^, whereas contact-based approaches such as actigraphy and consumer-wearables are second only to PSG in accuracy. Our study therefore, reinforces the utility of consumer wearables as a low-cost yet objective source of sleep metrics in population cohort studies.

Beyond comparisons with the usual clinical health markers, our study provides a novel demonstration of the utility of wearable-derived sleep data in the study of biological aging. We showed that in a normal free-living cohort, individuals with short habitual sleep duration experienced premature telomere attrition. Previous studies of this phenomenon have either used sleep questionnaires^33,46–48^ or cohorts of sleep disorder patients^49,50^. As premature telomere shortening has been linked to the early onset of various age-related diseases^51^ and all-cause mortality^52,53^, new evidence on the link between insufficient sleep and accelerated aging such as this are vital in helping shape public policy (e.g., later school start times, altered work hours and schedules, etc.) and to promote healthier sleep habits among the public. This finding is especially relevant to Singapore, a developed nation whose citizenry is among the most sleep-deprived in the world^41,54^. We did not identify in our study statistically significant shorter LTL among volunteers with long wearable-derived TST. This could be due to the small numbers of volunteers who are long sleepers. In fact, only one volunteer had wearable-derived TST exceeding 10 h. Our use of (1) wearables for sleep tracking and (2) WGS for LTL estimation in this study, demonstrate the versatility that emerging technologies can bring to population cohort studies by providing added behavioral and phenotypic data beyond their primary functions.

In summary, our study has demonstrated various aspects in which sleep metrics from wearables can be used in cohort studies. Apart from comparing wearable-derived and self-reported sleep metrics, our work has shown that wearables can be used to study how sleep relates to demographic, socioeconomic and lifestyle factors, as well as various markers of health and aging. The increasing ubiquity of wearables and other forms of digital health, represent a rich source of behavioral data that can be tapped by investigators running cohort studies. Beyond the use of wearables as study-provided devices, a BYOD (bring your own device) model, where participants share data from their own wearables with investigators through application programming interfaces (APIs), is also possible. This is particularly attractive as the BYOD model allows for much longer tracking durations with minimal incremental cost. At the same time, the use of wearables and other digital health devices in population health studies can catalyze further development of digital applications that promote healthy behavior, including sleep habit.

## Methods

### Study volunteers and ethics statement

Volunteers responding to print advertisements were recruited as part of the SingHEART/Biobank study using a protocol and written informed consent form approved by the SingHealth Centralized Institutional Review Board (ref: 2015/2601). The cohort’s details and its inclusion criteria have been previously described^24^. Among others, volunteers underwent an activity tracking study using a consumer wearable (Fitbit Charge HR) and detailed clinical profiling of various CVD risk markers (anthropometry, blood pressure, lipid panel, FBG, etc.). In addition, DNA was extracted from volunteer whole blood samples for molecular studies. After evaluation for completeness of sleep and activity tracking data, and the removal of subjects with extreme outlier activity metrics, 482 volunteers were included in this study. The sample size is comparable to, or exceeds that of similar studies^18,19^.

### Processing of wearable sleep metrics

For each volunteer, we extracted their Fitbit data (activity, heart rate and sleep) using the Fitbit Web API (https://dev.fitbit.com/reference/web-api/quickstart/). Data completeness was evaluated by availability of HR data, and days with no intraday steps were excluded^24^. We considered days with at least 20 h of data to be complete, and only volunteers with at least three data-complete days were included. Detailed sleep tracking data from Fitbit was obtained in the JSON (JavaScript Object Notation) format, and processed using an R. For each day, we summed the duration of all sleep sessions starting between 8 PM and 8 AM. We then averaged daily sums for each volunteer to obtain the TST. Sleep hour was determined by calculating the average start time of sleep sessions occurring between 8 PM and 8 AM with duration more or equal to 3 h. Wake hour was determined by averaging the end time of sleep sessions. SE was computed in a similar fashion, except that for each day, the average SE of sleep sessions was obtained. In addition, we estimated the number of nocturnal awakenings by averaging daily total wake counts.

### Questionnaires

The volunteer recruitment process included the administration of several questionnaires. This included the SingHEART patient questionnaire which covered demographics, socioeconomic factors, medical history, smoking history, alcohol consumption patterns, exercise and dietary habits. The General Practice Physical Activity Questionnaire (GPPAQ) was used to estimate physical activity levels as previously described^24^. Furthermore, the Pittsburgh Sleep Quality Index (PSQI) questionnaire, which assesses sleep quality and disturbances over a one-month time interval, was also administered^27^. Volunteers were asked about their sleep habits, for example their bed time, hours of sleep per night, sleep trouble and sleep quality. The PSQI contains 19 self-rated questions and 5 questions rated by the bed partner or roommate (if one is available). These 19 self-rated items are then combined to produce seven component scores, each of which has a range of 0–3. Components 3 and 4, as well as Question 5b were examined in this study due to their relevance to our wearable-derived sleep metrics (TST, SE and nocturnal awakenings respectively).

### Association tests

Multiple linear regression analyses described in this study were conducted using the GLM (generalized linear model) function in R and Gaussian error distribution was used. When gender was considered as a covariate, the female gender was set as the reference level. Whereas the following are the reference level for each of the socioeconomic factor when it was set as a covariate: “Public-housing” for residence type; “Others” for education level and “Manual-labor” for occupation type. For lifestyle factors, the reference level for alcohol and caffeine, tea and green tea consumption is “No”; the reference levels for smoking and exercise status are “Ex-smoker” and “Never/hardly” respectively.

For linear regression analysis between wearable-derived sleep metrics and CVD risk markers, three models were used. Age, gender, ethnicity, daily step counts were included as covariates. For Model 1 and Model 2 which considered TST and SE-based sleep metric, the model is, respectively, Marker ~ Age + Gender + Ethnicity + AverageDailyTotalSteps + TST and Marker ~ Age + Gender + Ethnicity + AverageDailyTotalSteps + SE. Whereas for Model 3 which includes both TST and SE, the model is Marker ~ Age + Gender + Ethnicity + AverageDailyTotalSteps + TST + SE. For linear regression analysis between wearable-derived TST metrics and socioeconomic factors, the following model was used: TST ~ Age + Gender + Ethnicity + SocioeconomicFactor. In addition, linear regression analysis between wearable-derived TST metrics and LTL was done by using this model: LTL ~ Age + Gender + Ethnicity + BMI + TST. For analysis of interactions between wearable-derived sleep metrics and age or gender, the model used was LTL ~ TST * Age + Gender + Ethnicity + BMI and LTL ~ TST * Gender + Age + Ethnicity + BMI, respectively.

### DNA extraction

Genomic DNA was extracted from volunteer whole blood specimens using the Chemagic DNA blood kit (Perkin Elmer, MA) following manufacturer’s protocol. The quality and quantity of extracted genomic DNA were assessed using LabChip DS (Perkin Elmer).

### Telomere length estimation

To estimate LTL from WGS data (WGS-LTL), we used the Telomerecat^31^ program, which does so by calculating the ratio between read-pairs completely mapping to the telomere and those that span the telomere boundary. As part of the SingHEART/Biobank study, we had sequenced the genomes of 546 volunteers (of which 175 overlapped with this study) at a target depth of 30×. The sequencing was performed by commercial sequencing providers using the Illumina Hiseq X platform. Telomerecat was used to estimate WGS-LTL for this dataset. Briefly raw sequencing reads in FASTQ files were aligned to the human reference genome (hs37d5) using BWA-MEM version 0.7.12^55^. The resulting BAM files were further processed using Sambamba version 0.5.8^56^ to sort the reads and flag duplicates. Telomerecat was run in two steps. First, the *bam2telbam* command was run on individual BAM files to generate *telbam* files, which are small BAM files containing only sequencing reads relevant to LTL estimation. The *telbam2length* command was then executed to generate LTL estimates for the entire 546 set of *telbam* files. Finally, we used linear regression to correct for different sequencing runs. BAM files containing telomeric and subtelomeric sequencing reads are available in the European Nucleotide Archive (ENA, accession number PRJEB29577). WGS data for the individuals are deposited in the European Genome-phenome Archive (EGA, accession number EGAS00001003570) and are available subject to Data Access Committee (DAC) approval.

### Telomere length measurement by qPCR

We used the qPCR method described by Cawthon^57,58^ to estimate LTL. The qPCR experiments were performed by operators blinded to participant characteristics. Briefly, two primer pairs are used, with one targeting telomere repeats (T) and another targeting a *36B4*, a known single-copy gene (S). For a given sample, the ratio between T and S amplification products (T/S ratio) is calculated. This T/S ratio correlates with telomere length, and the relative difference in T/S ratio between samples is proportional to the relative difference in their telomere lengths. Experimental details are as follows: Each genomic DNA sample was normalized to 35 ng/μL. Sequences of T and S primers and their final concentration are provided in Supplementary Table 6. A reference DNA sample (Promega, cat. no.: G1521) was diluted serially threefold from 109.0 ng/μL to 36.3, 12.1, 4.0, and 1.3 ng/μL to generate a standard curve. All samples were run in triplicate and the telomere and *36B4* PCR performed on two separate plates in identical well positions. Each reaction mix was prepared in 10 μL containing 1× PowerUp SYBR Green Master Mix (Applied Biosystems), 270–300 μM forward and 500–900 nM reverse primers and 35 ng gDNA or reference DNA. All PCRs were performed on StepOnePlus Real Time PCR system (Applied Biosystems) and began with Stage 1: 2 min at 50 °C, 2 min at 95 °C; Stage 2: 40 cycles of 15 s at 95 °C, 15 s of 54 °C (Telomere PCR) or 58 °C (*36B4* PCR), 1 min of 72 °C with signal acquisition. The T and S concentrations were interpolated using the standard curve and the averages of T and S concentrations were calculated from the triplicates. Any outliers were removed or repeated. Inter-plate variability was controlled using a normalization factor derived from a control sample (Promega, cat. no.: G3041) running in triplicates in each run. All T and S concentrations were corrected using the normalization factor. As the experiment was conducted in two batches, linear regression was used to adjust for batch effects in the T and S concentrations prior to calculating the final T/S ratios.

### Statistics and reproducibility

All statistical analyses in this study were performed using the R statistical environment. Correlations between continuous values (e.g., wearable-derived TST) and discrete questionnaire responses (e.g., PSQI scores) were calculated as Spearman’s rank correlation coefficients (denoted as *r*_s_), whereas correlations between pairs of continuous values were calculated as Pearson correlation coefficients (denoted as *r*_p_). The relationship between TST and LTL was explored in two separate cohorts. First, a discovery cohort comprising 175 individuals with LTL estimated using WGS and second, a validation cohort comprising 305 individuals with LTL estimated using qPCR. The validation cohort, apart from demonstrating the reproducibility of the observation made in the discovery cohort, also showed that the relationship was present regardless of method used to estimate LTL (WGS or qPCR).

### Reporting summary

Further information on research design is available in the Nature Research Reporting Summary linked to this article.

## Supplementary information

Supplementary Information

Description of Additional Supplementary Files

Reporting Summary

## Data Availability

Study participant characteristics (used to generate Table 1) are available in Supplementary Data 1 and Figshare (10.6084/m9.figshare.7835378). All data (including raw wearable metrics) are available in Supplementary Data 2 and Figshare (10.6084/m9.figshare.7835393). Quality control metrics (standard curves, amplification efficiencies, IC values, etc.) for qPCR assays are available in Supplementary Data 3 and Figshare (10.6084/m9.figshare.9042542).

## Code availability

R code used in the analyses and figures are available in Supplementary Data 2 and via Figshare (10.6084/m9.figshare.7835393).
