# Supplementary material for: Digital phenotyping by consumer wearables identifies sleep-associated markers of cardiovascular disease risk and biological aging
Source: Commun Biol. 2019 Oct 4;2:361. doi: 10.1038/s42003-019-0605-1 (PMC6778117; doi:10.1038/s42003-019-0605-1)
Supplement: Supplementary file 1 — Supplementary Information [file 42003_2019_605_MOESM1_ESM.pdf]

Supplementary Information

Supplementary Figures

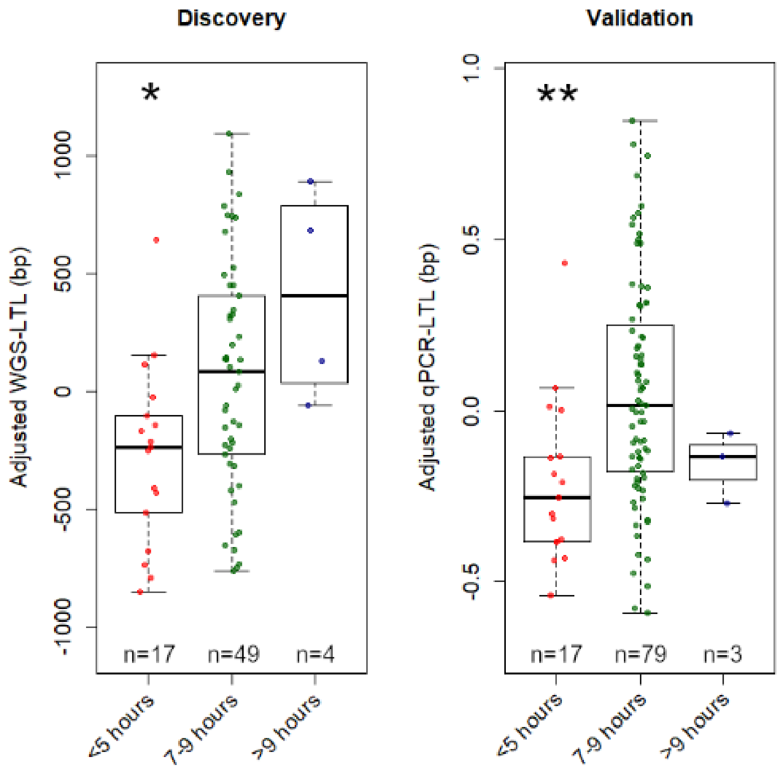

Supplementary Figure 1. Adjusted WGS-LTL and Adjusted qPCR-LTL of volunteers with insufficient (< 5 hours), adequate (> 7 hours but <= 9 hours) and long (> 9 hours) of TST. Adequate sleep group further stratified into two groups; adequate sleep and long sleep, with adequate sleep group set as reference. Asterisks denote significance of component score in linear model compared to reference score of 0. ** = p < 0.01, *** = p < 0.001. LTL = leukocyte telomere length; WGS-LTL = LTL estimated using whole-genome sequencing; qPCR-LTL = LTL estimated using quantitative PCR; TST = total sleep time; bp = base pairs; T/S = T/S ratio. All LTL values are adjusted for age, gender, ethnicity and BMI.

Supplementary Tables

Supplementary Table 1. Summary of multiple linear regression results for socioeconomic factors and sleep metrics, wearable-derived TST and self-reported TST. β-values, 95% confidence interval, P-values and standard error are shown. Highlighted cells have $p < 0.05$. The reference level in each model is stated as "ref" and highlighted in grey.

**Wearable-derived TST**				
**Socioeconomic factor**		**β (95% CI)**	**p**	**Std. Error**
**Occupation type**	**Manual labor**	ref		
	**Service Industry**	30.944 (0.811 to 61.077)	0.045	15.374
	**Unemployed or retired**	31.652 (6.363 to 56.941)	0.015	12.903
	**Office work, professionals**	24.768 (1.554 to 47.982)	0.037	11.844
**Residence type**	**Public-housing**	ref		
	**Private-housing**	14.606 (2.403 to 26.808)	0.019	6.226
	**Others**	25.199 (-1.451 to 51.848)	0.064	13.957
**Education levels**	**Others**	ref		
	**University and above**	-4.177 (-6.903 to 15.256)	0.460	5.653
**Income levels**	**Numeric(Income.levels)**	-0.424 (-3.249 to 2.400)	0.769	1.441
**Self-reported TST**				
**Socioeconomic factor**		**β (95% CI)**	**p**	**Std. Error**
**Occupation type**	**Manual labor**	ref		
	**Service Industry**	0.379 (-0.145 to 0.902)	0.157	0.267
	**Unemployed or retired**	0.054 (-0.385 to 0.493)	0.809	0.224
	**Office work, professionals**	-0.136 (-0.540 to 0.267)	0.508	0.206
**Residence type**	**Public-housing**	ref		
	**Private-housing**	0.050 (-0.163 to 0.263)	0.646	0.109
	**Others**	-0.120 (-0.586 to 0.346)	0.615	0.238
**Education levels**	**Others**	ref		
	**University and above**	-0.129 (-0.322 to 0.063)	0.189	0.098
**Income levels**	**Numeric(Income.levels)**	-0.046 (-0.095 to 0.003)	0.065	0.025

Supplementary Table 2. Summary of multiple linear regression results for lifestyle factors and sleep metrics, wearable-derived TST and self-reported TST. β-values, 95% confidence interval, P-values and standard error are shown. Highlighted cells have $p < 0.05$. The reference level in each model is stated as "ref" and highlighted in grey.

**Wearable-derived TST**				
**Lifestyle factor**		**β (95% CI)**	**p**	**Std. Error**
**Exercise.Weekly**	**Never/hardly**	ref		
	**Often/always**	-0.530 (-12.028 to 10.967)	0.928	5.866
**Smoking**	**Ex-smoker**	ref		
	**Yes**	-2.098 (-48.715 to 44.519)	0.930	23.785
	**No**	-6.701 (-46.359 to 32.957)	0.741	20.234
**Alcohol**	**No**	ref		
	**Yes**	19.247 (8.008 to 30.486)	8.54E-04	5.734
**Hard Liquor**	**No**	ref		
	**Yes**	28.049 (10.403 to 45.695)	0.002	9.00
**Beer**	**No**	ref		
	**Yes**	17.710 (3.609 to 31.811)	0.014	7.194
**Red Wine**	**No**	ref		
	**Yes**	19.219 (5.207 to 33.232)	0.008	7.149
**White Wine**	**No**	ref		
	**Yes**	10.457 (-6.360 to 27.273)	0.224	8.58
**Sparkling Wine**	**No**	ref		
	**Yes**	2.630 (-18.702 to 23.961)	0.809	10.884
**Caffeine**	**No**	ref		
	**Yes**	2.442 (-9.505 to 14.390)	0.689	6.10
**Tea**	**No**	ref		
	**Yes**	3.800 (-6.949 to 14.550)	0.489	5.485
**Green Tea**	**No**	ref		
	**Yes**	10.195 (-0.322 to 20.711)	0.058	5.366
**Vegetable servings**		2.233 (-3.331 to 7.796)	0.432	2.839
**Fruit servings**		3.085 (-4.340 to 10.509)	0.416	3.788

Self-reported TST				
**Lifestyle factor**		**β (95% CI)**	**p**	**Std. Error**
**Exercise.Weekly**	**Never/hardly**	ref		
	**Often/always**	0.096 (-0.103 to 0.295)	0.345	0.102
**Smoking**	**Ex-smoker**	ref		
	**Yes**	-0.054 (-0.863 to 0.754)	0.895	0.413
	**No**	-0.003 (-0.691 to 0.685)	0.99	0.351
**Alcohol**	**No**	ref		
	**Yes**	0.033 (-0.166 to 0.231)	0.748	0.101
**Hard Liquor**	**No**	ref		
	**Yes**	-0.128 (-0.440 to 0.184)	0.422	0.16
**Beer**	**No**	ref		
	**Yes**	-0.032 (-0.284 to 0.220)	0.805	0.129
**Red Wine**	**No**	ref		
	**Yes**	0.043 (-0.200 to 0.285)	0.731	0.124
**White Wine**	**No**	ref		
	**Yes**	-0.034 (-0.329 to 0.260)	0.819	0.15
**Sparkling Wine**	**No**	ref		
	**Yes**	0.005 (-0.369 to 0.379)	0.979	0.191
**Caffeine**	**No**	ref		
	**Yes**	0.056 (-0.151 to 0.263)	0.597	0.106
**Tea**	**No**	ref		
	**Yes**	0.107 (-0.080 to 0.293)	0.262	0.095
**Green Tea**	**No**	ref		
	**Yes**	-0.050 (-0.233 to 0.133)	0.592	0.093
**Vegetable servings**		0.024 (-0.073 to 0.121)	0.629	0.049
**Fruit servings**		-0.004 (-0.135 to 0.127)	0.950	0.067

Supplementary Table 3. Association between wearable-derived sleep metrics and CVD risk markers – Model 3. Model 3 = TST + SE. This model include age and gender as covariates. Highlighted cells are statistically significant ($p < 0.05$). BMI = body mass index; WC = waist circumference; WHtR = waist-to-height ration; BFP = body fat percentage; SMP = skeletal muscle percentage; SBP = systolic blood pressure; DBP = diastolic blood pressure; TotalChol = total cholesterol; LDL = low-density lipoprotein; HDL = high-density lipoprotein; TG = triglycerides; FBG = fasting blood glucose; TST = total sleep time; SE = sleep efficiency.

Wearable-derived TST and SE				
Marker	Model 3[c]			
	Wearable-derived TST		Wearable-derived SE	
	β (95% CI)	p	β (95% CI)	p
BMI	-5.044E-03 (-1.048E-02 to -3.967E-04)	0.070	-9.662E-02 (-2.010E-01 to 7.774E-03)	0.070
WC	3.877E-03 (-1.225E-02 to -2.000E-02)	0.638	-4.20E-01 (-7.291E-01 to -1.103E-01)	0.008
WHtR	-2.120E-05 (-1.184E-04 to 7.602E-05)	0.669	-2.464E-03 (-4.329E-03 to -5.981E-04)	0.010
RestingHR	-1.455E-02 (-2.439E-02 to -4.703E-03)	0.004	1.095E-02 (-1.779E-01 to 1.998E-01)	0.910
SBP	-7.654E-03 (-3.275E-02 to 1.744E-02)	0.550	-1.609E-01 (-6.425E-01 to 3.207E-01)	0.513
DBP	-8.310E-03 (-2.699E-02 to 1.037E-02)	0.384	9.245E-03 (-3.492E-01 to 3.677E-01)	0.960
TotalChol	-1.551E-03 (-3.005E-03 to -9.700E-05)	0.037	8.739E-03 1.916E-02 to 3.664E-02)	0.540
LDL	-1.344E-03 (-2.675E-03 to -1.301E-05)	0.048	6.179E-03 (-1.941E-02 to 3.177E-02)	0.636
HDL	-1.609E-04 (-6.467E-04 to 3.249E-04)	0.517	9.838E-03 5.171E-04 to 1.916E-02)	0.039
TG	-1.486E-04 (-1.184E-03 to 8.869E-04)	0.779	-8.470E-03 (-2.834E-02 to 1.140E-02)	0.404
FBG	-2.235E-04 (-1.161E-03 to 7.135E-04)	0.640	-1.881E-03 (-1.981E-02 to 1.605E-02)	0.837
BFP	-9.121E-03 (-2.021E-02 to 1.973E-03)	0.108	-1.501E-01 (-3.622E-01 to 6.205E-02)	0.166
SMP	5.859E-03 (-8.088E-04 to 1.253E-02)	0.086	8.835E-02 (-3.915E-02 to 2.159E-01)	0.175

[c]Marker~Age+Gender+Ethnicity+AverageDailyTotalSteps+Wearable-derived TST+ Wearable-derived SE

Supplementary Table 4. Association between self-reported (PSQI-derived) sleep metrics and CVD risk markers. Model 1 = TST only, Model 2 = SE only, Model 3 = TST + SE. All models include age and gender as covariates. Highlighted cells are statistically significant ($p < 0.05$). BMI = body mass index; WC = waist circumference; WHtR = waist-to-height ration; BFP = body fat percentage; SMP = skeletal muscle percentage; SBP = systolic blood pressure; DBP = diastolic blood pressure; TotalChol = total cholesterol; LDL = low-density lipoprotein; HDL = high-density lipoprotein; TG = triglycerides; FBG = fasting blood glucose; TST = total sleep time; SE = sleep efficiency.

Self-reported TST and SE								
Marker	Model 1[a]		Model 2[b]		Model 3[c]			
	Self-reported TST		Self-reported SE		Self-reported TST		Self-reported SE	
	β (95% CI)	p	β (95% CI)	p	β (95% CI)	p	β (95% CI)	p
BMI	-1.909E-01 (-4.977E-01 to 1.159E-01)	0.223	1.866E-03 (-3.104E-02 to 3.477E-02)	0.912	-2.541E-01 (-6.009E-01 to 9.272E-02)	0.152	1.454E-02 (-2.261E-02 to 5.168E-02)	0.443
WC	-6.062E-02 (-9.708E-01 to 8.496E-01)	0.896	1.879E-02 (-7.869E-02 to 1.163E-01)	0.706	-1.817E-01 (-1.211 to 8.477E-01)	0.730	2.785E-02 (-0.082 to 1.381E-01)	0.621
WHtR	-3.878E-04 (-5.877E-03 to 5.101E-03)	0.890	1.482E-04 (-4.396E-04 to 7.360E-04)	0.621	-1.318E-03 (-7.524E-03 to 4.889E-03)	0.678	2.139E-04 (-4.508E-04 to 8.787E-04)	0.529
RestingHR	-1.199E-01 (-6.762E-01 to 4.365E-01)	0.673	-8.051E-04 (-6.040E-02 to 5.879E-02)	0.979	-1.486E-01 (-7.779E-01 to 4.807E-01)	0.644	6.606E-03 (-6.079E-02 to 7.400E-02)	0.848
SBP	2.441E-01 (-1.163 to 1.652)	0.734	8.678E-02 (-6.378E-02 to 2.373E-01)	0.259	-1.700E-01 (-1.760 to 1.420)	0.834	9.526E-02 (-7.504E-02 to 2.656E-01)	0.273
DBP	6.908E-02 (-9.785E-01 to 1.117)	0.897	3.353E-02 (-7.863E-02 to 1.457E-01)	0.558	-9.790E-02 (-1.282 to 1.087)	0.871	3.841E-02 (-8.846E-02 to 1.653E-01)	0.553
TotalChol	1.775E-02 (-6.410E-02 to 9.960E-02)	0.671	1.971E-04 (-8.571E-03 to 8.965E-03)	0.965	2.157E-02 (-7.101E-02 to 1.142E-01)	0.648	-8.786E-04 (-1.079E-02 to 9.037E-03)	0.862
LDL	4.922E-03 (-6.990E-02 to 7.974E-02)	0.898	-3.992E-04 (-8.411E-03 to 7.613E-03)	0.922	8.523E-03 (-7.618E-02 to 9.323E-02)	0.844	-8.255E-04 (-9.896E-03 to 8.245E-03)	0.859
HDL	7.377E-04 (-2.661E-02 to 2.808E-02)	0.958	-3.224E-04 (-3.251E-03 to 2.606E-03)	0.829	2.731E-03 (-2.820E-02 to 3.366E-02	0.863	-4.586E-04 (-3.771E-03 to 2.854E-03)	0.786
TG	7.355E-03 (-5.071E-02 to 6.542E-02)	0.804	2.585E-03 (-3.630E-03 to 8.800E-03)	0.415	-4.960E-03 (-7.060E-02 -6.068E-02)	0.882	2.833E-03 (-4.197E-03 to 9.863E-03)	0.430
FBG	-3.777E-02 (-9.001E-02 to 1.448E-02)	0.157	-3.478E-03 (-9.068E-03 to 2.112E-03)	0.223	-2.889E-02 (-8.794E-02 to 3.015E-02)	0.338	-2.042E-03 (-8.356E-03 to 4.272E-03)	0.526
BFP	3.182E-01 (-3.036E-01 to 9.399E-01)	0.316	3.759E-02 (-2.907E-02 to 1.042E-01)	0.270	1.985E-01 (-5.031E-01 to 9.001E-01)	0.579	2.775E-02 (-4.748E-02 to 1.030E-01)	0.470
SMP	-2.138E-01 (-5.875E-01 to 1.600E-01)	0.263	-1.723E-02 (-5.733E-02 to 2.287E-02)	0.400	-1.774E-01 (-5.993E-01 to 2.445E-01)	0.410	-8.437E-03 (-5.368E-02 to 3.680E-02)	0.715

[a]Marker~Age+Gender+Ethnicity+AverageDailyTotalSteps+PSQI-derived TST
[b]Marker~Age+Gender+Ethnicity+AverageDailyTotalSteps+PSQI-derived SE
[c]Marker~Age+Gender+Ethnicity+AverageDailyTotalSteps+PSQI-derived TST+PSQI-derived SE

Supplementary Table 5. Analysis of interaction between wearable-derived sleep metrics and age or gender with CVD risk marker. Model 1 = TST*Age, Model 2 = TST*Gender, Model 3 = SE*Age, Model 4 = SE*Gender. β-values, 95% confidence interval, and P-values are shown. Highlighted cells are statistically significant ($p < 0.05$). BMI = body mass index; WC = waist circumference; WHtR = waist-to-height ration; BFP = body fat percentage; SMP = skeletal muscle percentage; SBP = systolic blood pressure; DBP = diastolic blood pressure; TotalChol = total cholesterol; LDL = low-density lipoprotein; HDL = high-density lipoprotein; TG = triglycerides; FBG = fasting blood glucose; TST = total sleep time; SE = sleep efficiency.

Interactions								
Marker	Model 1[a]		Model 2[b]		Model 3[c]		Model 4[d]	
	Wearable-derived TST*Age		Wearable-derived TST*Gender		Wearable-derived SE*Age		Wearable-derived SE*Gender	
	β (95% CI)	p	β (95% CI)	p	β (95% CI)	p	β (95% CI)	p
BMI	-1.148E-04 (-5.839E-04 to 3.541E-04)	0.632	8.588E-04 (-9.799E-03 to 1.152E-02)	0.875	2.828E-03 (-5.408E-03 to 1.106E-02)	0.501	1.415E-01 (-6.562E-02 to 3.487E-01)	0.181
WC	2.320E-04 (-1.164E-03 to 1.628E-03)	0.745	-3.518E-03 (3.523E-02 to 2.819E-02)	0.828	4.339E-03 (-2.000E-02 to 2.868E-02)	0.727	1.757E-01 (-4.373E-01 to 7.887E-01)	0.545
WHtR	3.870E-07 (-8.029E-06 to 8.802E-06)	0.928	-2.666E-05 (-2.178E-04 to 1.645E-04)	0.785	3.809E-05 (-1.086E-04 to 1.848E-04)	0.611	1.091E-03 (-2.605E-03 to 4.787E-03)	0.563
RestingHR	3.849E-04 (-4.605E-04 to 1.230E-03)	0.373	-9.519E-03 (-2.872E-02 to 9.679E-03)	0.332	2.426E-04 (-1.475E-02 to 1.523E-02)	0.975	1.140E-03 (-3.764E-01 to 3.787E-01)	0.995
SBP	2.425E-04 (-1.915E-03 to 2.401E-03)	0.826	-3.022E-02 (-7.916E-02 to 1.873E-02)	0.227	2.763E-02 (-1.018E-02 to 6.545E-02)	0.153	3.149E-02 (-9.231E-01 to 9.861E-01)	0.948
DBP	2.320E-04 (-1.651E-03 to 1.560E-03)	0.956	-4.396E-03 (-4.086E-02 to 3.207E-02)	0.813	1.027E-02 (-1.793E-02 to 3.847E-02)	0.476	-1.657E-01 (-8.763E-01 to 5.450E-01)	0.648
TotalChol	-4.568E-05 (-4.221E-05 to 2.074E-04)	0.195	-2.630E-04 (-3.103E-03 to 2.577E-03)	0.856	-1.337E-04 (2.339E-03 to 2.071E-03)	0.905	-1.009E-02 (-6.562E-02 to 4.545E-02)	0.722
LDL	5.182E-05 (-6.235E-05 to 1.660E-04)	0.374	-4.656E-04 (-3.065E-03 to 2.133E-03)	0.726	-6.107E-04 (-2.625E-03 to 1.403E-03)	0.553	-6.623E-03 (-5.752E-02 to 4.42702)	0.799
HDL	9.136E-06 (-3.280E-05 to 5.107E-05)	0.67	1.077E-04 (-8.448E-04 to 1.060E-03)	0.825	3.292E-04 (-4.037E-04 to 1.062E-03)	0.379	-3.070E-03 (-2.155E-02 to 1.541E-02)	0.745
TG	4.524E-05 (-4.373E-05 to 1.342E-04)	0.32	4.301E-04 (-1.592E-03 to 2.453E-03)	0.677	4.340E-04 (-1.128E-03 to 1.996E-03)	0.586	1.200E-03 (-3.817E-02 to 4.057E-02)	0.952
FBG	-7.938E-06 (-8.809E-05 to 7.221E-05)	0.846	-1.099E-03 (-2.926E-03 to 7.281E-04)	0.239	2.434E-04 (-1.166E-03 to 1.652E-03)	0.735	2.594E-02 (-9.558E-03 to 6.144E-02)	0.153
BFP	-5.733E-04 (-1.533E-03 to 3.866E-04)	0.242	2.926E-03 (-1.880E-02 to 2.466E-02)	0.792	1.161E-02 (-5.058E-03 to 2.828E-02)	0.173	-3.723E-02 (-4.586E-01 to 3.841E-01)	0.863

SMP	6.517E-04<br>(7.696E-05 to 1.226E-03)	0.027	-2.819E-03<br>(-1.588E-02 to 1.024E-02)	0.672	-4.432E-03<br>(-1.447E-02 to 5.603E-03)	0.387	-3.693E-03<br>(-2.570E-01 to 2.497E-01)	0.977

[a]Marker~Wearable-derived TST*Age+Gender+Ethnicity+AverageDailyTotalSteps
[b]Marker~Wearable-derived TST*Gender+Age+Ethnicity+AverageDailyTotalSteps
[c]Marker~Wearable-derived SE*Age+Gender+Ethnicity+AverageDailyTotalSteps
[d]Marker~Wearable-derived SE*Gender+Age+Ethnicity+AverageDailyTotalSteps

Supplementary Table 6. T and S primer sequences and their final concentrations.

Primer	Oligonucleotide Sequence	Final Concentration (nM)
tel1	5'-GGTTTTTGAGGGTGAGGGTGAGGGTGAGGGTGAGGGT-3'	270
tel2	5'-TCCCGACTATCCCTATCCCTATCCCTATCCCTATCCCTA-3'	900
36B4d	5'-CAGCAAGTGGGAAGGTGTAATCC-3'	300
36B4u	5'-CCCATTCTATCATCAACGGGTACAA-3'	500
