# Supplementary material for: Digital phenotyping by consumer wearables identifies sleep-associated markers of cardiovascular disease risk and biological aging
Source: Commun Biol. 2019 Oct 4;2:361. doi: 10.1038/s42003-019-0605-1 (PMC6778117; doi:10.1038/s42003-019-0605-1)
Supplement: Supplementary file 2 — Description of Additional Supplementary Files [file 42003_2019_605_MOESM2_ESM.docx]

**Description of Additional Supplementary Files**

**Supplementary Data 1.** Characteristics of study participants. Available online at Figshare ([https://doi.org/10.6084/m9.figshare.7835378](https://doi.org/10.6084/m9.figshare.7835378%20)).

**Supplementary Data 2.** All data and R code used in this work. Available online at Figshare (<https://doi.org/10.6084/m9.figshare.7835393>).

**Supplementary Data 3.** QC metrics for the qPCR-based LTL assays. Available online at Figshare (<https://doi.org/10.6084/m9.figshare.9042542>).
