# Supplementary material for: Digital phenotyping by consumer wearables identifies sleep-associated markers of cardiovascular disease risk and biological aging
Source: Commun Biol. 2019 Oct 4;2:361. doi: 10.1038/s42003-019-0605-1 (PMC6778117; doi:10.1038/s42003-019-0605-1)
Supplement: Supplementary file 3 — Reporting Summary [file 42003_2019_605_MOESM3_ESM.pdf]

nature research

Corresponding author(s): Weng Khong LIM, Khung Keong YEO, Patrick TAN

Last updated by author(s): Aug 28, 2019

Reporting Summary

Nature Research wishes to improve the reproducibility of the work that we publish. This form provides structure for consistency and transparency in reporting. For further information on Nature Research policies, see Authors & Referees and the Editorial Policy Checklist.

Statistics

For all statistical analyses, confirm that the following items are present in the figure legend, table legend, main text, or Methods section.

n/a	Confirmed	
☐	☒	The exact sample size (*n*) for each experimental group/condition, given as a discrete number and unit of measurement
☒	☐	A statement on whether measurements were taken from distinct samples or whether the same sample was measured repeatedly
☐	☒	The statistical test(s) used AND whether they are one- or two-sided<br>*Only common tests should be described solely by name; describe more complex techniques in the Methods section.*
☐	☒	A description of all covariates tested
☒	☐	A description of any assumptions or corrections, such as tests of normality and adjustment for multiple comparisons
☐	☒	A full description of the statistical parameters including central tendency (e.g. means) or other basic estimates (e.g. regression coefficient) AND variation (e.g. standard deviation) or associated estimates of uncertainty (e.g. confidence intervals)
☐	☒	For null hypothesis testing, the test statistic (e.g. *F*, *t*, *r*) with confidence intervals, effect sizes, degrees of freedom and *P* value noted<br>*Give P values as exact values whenever suitable.*
☒	☐	For Bayesian analysis, information on the choice of priors and Markov chain Monte Carlo settings
☒	☐	For hierarchical and complex designs, identification of the appropriate level for tests and full reporting of outcomes
☐	☒	Estimates of effect sizes (e.g. Cohen's *d*, Pearson's *r*), indicating how they were calculated

Our web collection on statistics for biologists contains articles on many of the points above.

Software and code

Policy information about availability of computer code

Data collection	Fitbit raw data were obtained Fitbit using the Fitbit Web API (https://dev.fitbit.com/reference/web-api/quickstart).
Data analysis	All analyses in the study were performed in the open source R statistical environment. All data (including raw wearable metrics) and R code used in the analyses and figures are available in Supplementary Data 2 (Figshare URL: https://figshare.com/s/dc94f20b69e07ea83a88).

For manuscripts utilizing custom algorithms or software that are central to the research but not yet described in published literature, software must be made available to editors/reviewers. We strongly encourage code deposition in a community repository (e.g. GitHub). See the Nature Research guidelines for submitting code & software for further information.

Data

Policy information about availability of data

All manuscripts must include a data availability statement. This statement should provide the following information, where applicable:

- Accession codes, unique identifiers, or web links for publicly available datasets
- A list of figures that have associated raw data
- A description of any restrictions on data availability

All data (including raw wearable metrics) and R code used in the analyses and figures are available in Supplementary Data 2 (https://doi.org/10.6084/m9.figshare.7835393). BAM files containing telomeric and subtelomeric sequencing reads are available in the European Nucleotide Archive (ENA, accession number PRJEB29577). WGS data for the individuals are deposited in the European Genome-phenome Archive (EGA, accession number EGAS00001003570) and are available subject to Data Access Committee (DAC) approval.

Field-specific reporting

Please select the one below that is the best fit for your research. If you are not sure, read the appropriate sections before making your selection.

☒ Life sciences ☐ Behavioural & social sciences ☐ Ecological, evolutionary & environmental sciences

For a reference copy of the document with all sections, see nature.com/documents/nr-reporting-summary-flat.pdf

Life sciences study design

All studies must disclose on these points even when the disclosure is negative.

Sample size	The sample size (n = 482) was determined by the number of volunteers available with Fitibt data that mached our data inclusion criteria (see below). This sample size is comparable to, or exceeds the sample size used in similar studies [refs 18 and 19].
Data exclusions	Fitbit data: Data completeness was evaluated by the availability of HR data and days with no intraday steps were excluded (full details are available in [ref 24]). We considered days with at least 20 hours of data to be complete, and only volunteers with at least three data-complete days were included.
Replication	Relationship between wearable-derived TST and telomere length was identified on a discovery cohort of 175 individuals with WGS data. This relationship was replicated in a validation cohort comprising 305 individuals who had telomere length measured using qPCR.
Randomization	In the sleep <-> telomere length association analysis, allocation of participants into discovery (n = 175) and validation (n = 305) cohorts is random.
Blinding	For the qPCR telomere length assays, the operator was blinded to all participant phenotypes and characteristics (e.g. age, gender, sleep duration).

Reporting for specific materials, systems and methods

We require information from authors about some types of materials, experimental systems and methods used in many studies. Here, indicate whether each material, system or method listed is relevant to your study. If you are not sure if a list item applies to your research, read the appropriate section before selecting a response.

Materials & experimental systems

n/a	Involved in the study	
☒	☐	Antibodies
☒	☐	Eukaryotic cell lines
☒	☐	Palaeontology
☒	☐	Animals and other organisms
☐	☒	Human research participants
☒	☐	Clinical data

Methods

n/a	Involved in the study	
☒	☐	ChIP-seq
☒	☐	Flow cytometry
☒	☐	MRI-based neuroimaging

Human research participants

Policy information about [studies involving human research participants]()

Population characteristics	Available in Table 1 of manuscript. Participants responded to advertisements seeking normal volunteers for heart study. Inclusion criteria are:1. Aged between 21 and 69 years. 2. No personal medical history of myocardial infarction (MI), coronary artery disease (CAD), peripheral arterial disease (PAD), stroke, cancer, autoimmune/genetic disease, endocrine, disease, diabetes mellitus, psychiatric illness, asthma, or chronic lung disease and chronic infective disease. 3. No family medical history of cardiomyopathies
Recruitment	Participants responded to print advertisements seeking normal volunteers for heart study. There may be potential for self-selection as these volunteers would presumably be more conscious of their health. However, impact to results should be minimum as we observed no evidence that self-selection affects the two key variables; sleep duration and telomere length.
Ethics oversight	Volunteers were recruited as part of the SingHEART/Biobank study using a protocol and written informed consent form approved by the SingHealth Centralized Institutional Review Board (ref: 2015/2601).

Note that full information on the approval of the study protocol must also be provided in the manuscript.
